# Democratization of Copper Analysis in Grape Must Following a Polymer-Based Lab-on-a-Chip Approach

**DOI:** 10.1021/acsami.3c00395

**Published:** 2023-03-20

**Authors:** José Carlos Guirado-Moreno, Israel Carreira-Barral, Saturnino Ibeas, José M. García, Daniel Granès, Nicolas Marchet, Saúl Vallejos

**Affiliations:** † Departamento de Química, Facultad de Ciencias, Universidad de Burgos, Plaza Misael Bañuelos s/n, 09001 Burgos, Spain; ‡ Direction Générale, La Jasse de Maurin, Groupe ICV, 34970 Lattes, France

**Keywords:** copper detection, grape must, wine industry, sensory polymers, colorimetry, RGB parameters

## Abstract

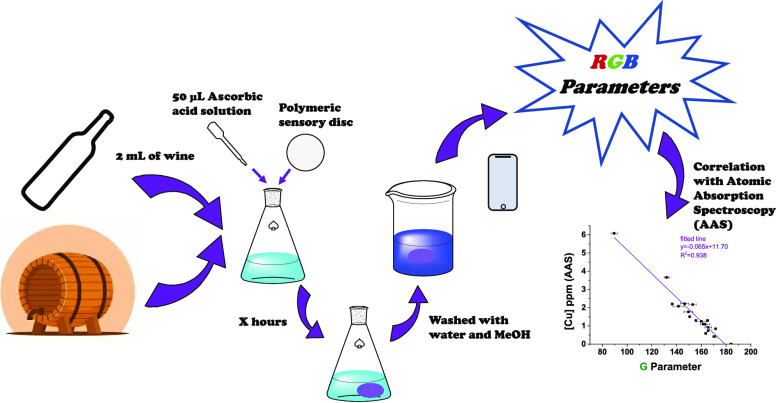

Quality control in the food industry is of the upmost importance from the food safety, organoleptic and commercial viewpoints. Accordingly, the development of *in situ*, rapid, and costless analytical tools is a valuable task in which we are working. Regarding this point, the copper content of grape must has to be determined by wineries along the wine production process. For this purpose, grape must samples are sent to laboratories where the copper content is measured usually by flame atomic absorption spectrometry or by inductively coupled plasma mass spectrometry. We herein propose a straightforward, rapid, and inexpensive methodology based both on a film-shaped colorimetric polymer sensor and a smartphone method that at the same time can be used by unskilled personnel. The sensory polymer films change their color upon dipping them on the grape must, and the color evolution is analyzed using the digital color parameters of a picture taken to the film with a smartphone. Furthermore, the analytical procedure is automatically carried out by a smartphone app. The limit of detection of copper of the polymer sensor is 0.08 ppm. Following this approach, 18 production samples coming from the French Groupe ICV company were studied. The copper content of the samples was analyzed by the usual procedure carried out by the company (flame atomic absorption spectrometry) and by the method proposed in this work, ranging this content from 0.41 to 6.08 ppm. The statistical study showed that the results of both methods are fully consistent, showing the validity of the proposed method for the determination of copper in grape must within the frame of wine production wineries and industries.

## 1 Introduction

Quality control in processed food products is an issue both related to food safety and to the organoleptic perception by the final user. Accordingly, it is gaining increasing relevance yearly, both in final products and in the intermediate production stages.^1−5^ For example, a number of analyses are mandatory to pass specific controls and/or to determine the concentration of different markers in beverages such as wine and are related to different organoleptic properties, such as color, flavor, etc.

During the intermediate production processes, grape must contains between 120 and 250 g/L sugar and between 2.5 and 3.5 g/L mineral substances.^6−8^ Copper is a minority part of the latter, so the amount is quite low.^6^ On the one hand, its presence is essential to obtain quality wines since metallic cations such as copper, potassium, magnesium, iron, calcium, cobalt, and zinc are necessary for the proper alcoholic fermentation of sugars. On the other hand, high levels of them could generate problems to the beverage’s quality or safety. Metal tanks and pipes used in manufacturing or antifungal treatments in cultivation can increase the concentration of metals such as copper, giving rise to the so-called cupric cracks, which manifest as reddish-brown sediments. The cupric crack is especially important in white wines and is usually avoided by adding bentonite or arabic gum. However, these treatments are not applicable for copper concentrations above 1 mg/L.^9−13^

Among the most common techniques in wineries for copper quantification are flame atomic absorption spectrometry (FAAS) and inductively coupled plasma mass spectrometry (ICP-MS), which imply non-*in situ* analyses carried out by specialized personnel and with high-cost equipment.^14^ Smart polymers can offer more direct and simple solutions to this type of analysis. Smart polymers can respond to a stimulus with an action (reactive polymers) or with an alert (sensory polymers).^15^ The latter is based on the same concept as conventional colorimetric probes for anions^16,17^ or cations^18−20^ but provides great added value by dispensing with the handling of reagents and solvents and being able to work in completely aqueous media.^21−25^

In this work, we propose the democratization of this type of analysis of copper in grape must by using a film-shaped smart sensory polymer that selectively interacts with copper and generates a color change. Furthermore, our proposal combines the use of the film with a free smartphone application,^3^ which analyzes the RGB parameters of the formed color. The material is mainly based on commercially available monomers (99.5 mol %), combined with a small amount (0.5 mol %) of a sensory monomer based on the chemical structure of a well-known copper chelating agent as bicinchoninic acid (BCA).^26,27^

## 2 Experimental Section

### 2.1 Materials

All materials and solvents were commercially available and used as received unless otherwise indicated. The following materials and solvents were used: 1-vinyl-2-pyrrolidone (VP) (99%, Sigma-Aldrich), methyl methacrylate (MMA) (99%, Sigma-Aldrich), ethylene glycol dimethacrylate (E) (97.5%, Sigma-Aldrich), pH 5.00 citrate buffer (VWR), acetone (99%, VWR), zinc(II) nitrate hexahydrate (98%, Sigma-Aldrich), iron(III) nitrate nonahydrate (99%, Sigma-Aldrich), manganese(II) nitrate hexahydrate (98+%, Alfa Aesar), cobalt(II) nitrate hexahydrate (≥99%, Labkem), calcium nitrate tetrahydrate (≥99%, Sigma-Aldrich), mercury(II) nitrate (98%, Alfa Aesar), cadmium nitrate tetrahydrate (98.5%, Alfa Aesar), potassium nitrate (99+%, Sigma-Aldrich), lead(II) nitrate (≥99%, Fluka), iron(II) sulfate heptahydrate (99%, Sigma-Aldrich), magnesium nitrate hexahydrate (≥99%, Labkem), copper(II) sulfate pentahydrate (98%, Sigma-Aldrich), copper(I) iodide (99%, Riedel-de-Häen), nickel(II) nitrate hexahydrate (98.5%, Sigma-Aldrich), sodium nitrate (99%, LabKem), cesium nitrate (≥99%, Fluka), barium chloride dihydrate (99%, Labkem), ammonium nitrate (≥98%, Sigma-Aldrich), chromium(III) nitrate (98.5%, Alfa Aesar), rubidium nitrate (99.95%, Sigma-Aldrich), dysprosium(III) nitrate (99.9%, Alfa Aesar), lithium chloride (≥99%, Sigma-Aldrich), cerium(III) chloride tetrahydrate (≥99.99%, Sigma-Aldrich), zirconium(IV) chloride (98%, Alfa Aesar), lanthanum(III) nitrate hexahydrate (99.9%, Alfa Aesar), samarium(III) nitrate (99.9%, Alfa Aesar), aluminum(III) nitrate (≥98.9%, Sigma-Aldrich), silver(I) nitrate (≥99.9%, Sigma-Aldrich), neodymium(III) nitrate (99.9%, Alfa Aesar), strontium nitrate (99%, Sigma-Aldrich), potassium hydroxide (99%, VWR-Prolabo), hydrochloric acid (37%, VWR-Prolabo), ethanol (≥99.9%, VWR), methanol (≥99.8%, VWR), tetrahydrofuran (≥99.9%, VWR), ethyl acetate (≥99.9%, VWR), dimethylsulfoxide-*d*_6_ (99.9%, VWR), dimethylformamide (99.9%, Supelco), 4-aminostyrene (≥98%, TCI), *N*,*N*′-dicyclohexylcarbodiimide (DCC) (99%, Sigma-Aldrich), and bicinchoninic acid disodium salt (≥98%, TCI). Azo-bis-isobutyronitrile (AIBN, Sigma-Aldrich, 98%) was recrystallized twice from methanol.

The grape must samples were provided by the French company “Groupe ICV”, collected in late summer 2021, frozen and sent to the University of Burgos and Groupe ICV-Toulouges, to perform the copper analysis by two methodologies, i.e., using the sensory polymer (proposed method) and FAAS (reference method). Once thawed, sediments can be formed in grape must samples, so they were stabilized with sodium azide (NaN_3_) before the frozen process. More information about grape must samples can be found in the Supporting Information, Section S1.

### 2.2 Instrumentation and Methods

^1^H and ^13^C{^1^H} NMR spectra (Advance III HD spectrometer, Bruker Corporation, Billerica, Massachusetts, USA) were recorded at 300 MHz for ^1^H and 75 MHz for ^13^C using deuterated dimethylsulfoxide (DMSO-*d*_6_) at 25 °C as the solvent.

The polymers’ thermal and mechanical characterization were performed by (a) thermogravimetric analysis (Q50 TGA analyzer, TA Instruments, New Castle, DE, USA) with 10–15 mg of the sample under synthetic air and nitrogen atmosphere at 10 °C·min^–1^; (b) differential scanning calorimetry, with 10–15 mg of the sample under a nitrogen atmosphere at a heating rate of 10 °C·min^–1^ (Q200 DSC analyzer, TA Instruments, New Castle, DE, USA); and (c) tensile property analysis of the samples (5 × 9.44 × 0.122 mm at 1 mm·min^–1^) (Shimadzu EZ Test Compact Table-Top Universal Tester, Shimadzu, Kyoto, Japan).

Infrared spectra (FTIR) were recorded with an infrared spectrometer (FT/IR-4200, Jasco, Tokyo, Japan) with an ATR-PRO410-S single reflection accessory.

FAAS was performed with an atomic absorption spectrophotometer (GBC 933 AA, Gbc Scientific, Braeside, Australia).

Ultraviolet visible spectroscopy (UV–vis) spectra were recorded using a spectrophotometer (Hitachi U-3900, Hitachi, Tokyo, Japan). A rectangular 10 mm cuvette was used for the measurements, which were all conducted at 25 ± 0.1 °C.

The weight percentage of water taken up by the films upon soaking in pure water at 20 °C until reaching equilibrium (water-swelling percentage, WSP) was obtained from the weight of a dry sample film (ω_d_) and its water-swelled weight (ω_s_) using the following expression: WSP = 100 × [(ω_s_ – ω_d_)/ω_d_].

High-resolution mass spectrometry (HRMS) was carried out on an Agilent 1260 HPLC-Infinity coupled with a 6545 ESI-Q-TOF/MS system (Agilent Technologies, Palo Alto, CA, USA).

Digital photographs were taken with a Huawei p30 pro (Huawei, Shenzhen, China), placing the films within a homemade lightbox to reproduce always the same lighting conditions.^24^ The distance between the object and the smartphone was 13 cm. The G parameter of digital photographs was extracted using the smartphone app “Colorimetric Titration”.^28,29^

### 2.3 Synthesis of the Sensory Monomer **Mono-BCA**

**Figure 1:**
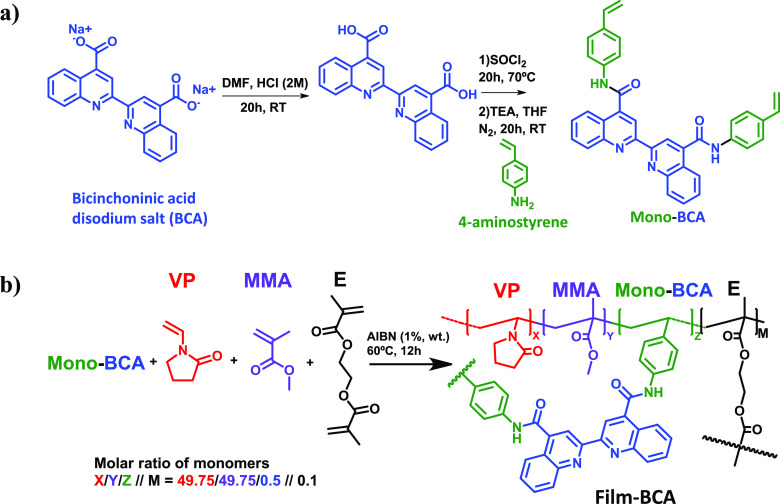
Synthetic route for (a) the sensory monomer **Mono-BCA** and (b) the film-shaped sensory polymer **Film-BCA**.

We prepared the sensory monomer **Mono-BCA** using bicinchoninic acid disodium salt as reactant. The reactions are depicted in Figure 1a, and the complete description of the synthetic procedure and the full characterization of intermediates and monomer can be found in the Supporting Information (Section S2).

### 2.4 Preparation of the Sensory Polymer Synthesis **Film-BCA**

We prepared the sensory film **Film-BCA** by thermally initiated bulk radical polymerization, as shown in Figure 1b.^30^ In a vial, two commercial monomers (VP and MMA), a cross-linker (ethylene glycol dimethacrylate, E), the synthesized sensory monomer (**Mono-BCA**), and DMSO (same volume as the sum of monomers) were mixed in a molar ratio of 49.75/49.75/0.5//0.1 (VP/MMA/**Mono-BCA**//E). Additionally, we added AIBN (1 wt %) as the radical thermal initiator. We injected the mixture into a mold (200 μm thickness) composed of two sealed silanized glasses and heated it overnight at 60 °C, where the polymerization took place under an oxygen-free atmosphere. Finally, we washed the resultant film-shaped material with water and methanol and cut it into 8 mm side squares with plastic scissors. The sensory material was stored in zip bags to keep it moist and to prevent it from drying out and cracking. Plastic or glass materials were used throughout the process to avoid contamination with copper. The FTIR, TGA, DSC, and PXRD patterns can be found in Section S3.

### 2.5 Preparation of the Solution to Record the Mass Spectrum of the 2:1 and 1:1 **Mono-BCA**:Cu(I) Complexes

First, a 10^–4^ M solution of **Mono-BCA** in DMSO was prepared. Second, a DMSO solution containing CuSO_4_·5H_2_O (10^–3^ M) and ascorbic acid (3.3 × 10^–3^ M) was prepared; ascorbic acid was required to reduce Cu(II) to Cu(I). Subsequently, 1.4 mL of the latter was added to 2.0 mL of the former while stirring. In this way, the solution contains an excess of Cu(I) (6.7 equiv), thus ensuring the formation of the complex. An aliquot of this solution was taken, and its mass spectrum (+ESI) was recorded. The spectrum and the magnification of the peaks corresponding to the 2:1 and 1:1 **Mono-BCA**:Cu(I) complexes are displayed in Section S4.

### 2.6 Selectivity Study

We conducted a selectivity study to evaluate the colorimetric response of **Film-BCA** with 30 different cations. Sensory squares were dipped into aqueous solutions of cations (5 × 10^–3^ M, distilled water) for 12 h. Finally, sensory squares were washed with water and methanol and were photographed together under the same light conditions.

### 2.7 Titration by UV–Vis of Cu(I) with **Mono-BCA**

A CuSO_4_·5H_2_O solution (1 × 10^–3^ M, DMSO) was titrated with a solution of **Mono-BCA** (1.04 × 10^–4^ M, DMSO), and the spectra were recorded at final copper concentrations ranging from 4.9 × 10^–6^ to 4.1 × 10^–4^ M, DMSO. The copper solution included 0.06 mg/mL ascorbic acid, necessary to reduce Cu(II) to Cu(I). UV–vis spectra were recorded at 25 C ± 0.1 °C using the following conditions: slit width = 2 nm; scan speed = 600 nm/min; step = 0.5 nm.

### 2.8 Titration of Cu(I) with **Film-BCA**

We carried out the experiment by immersing for 12 h **Film-BCA** sensory squares (8 mm side) in solutions containing 50 μL of a pH 5 buffered aqueous solution of ascorbic acid (0.08 g/mL) and 2 mL of a pH 5 buffered aqueous solution of Cu(II), with metal concentrations ranging from 0.5 to 10 ppm. After that, sensory squares were washed with water and photographed with the smartphone. The measurements were carried out in duplicate.

### 2.9 Detection and Quantification of Copper in Grape Musts with **Film-BCA**

**Figure 2:**
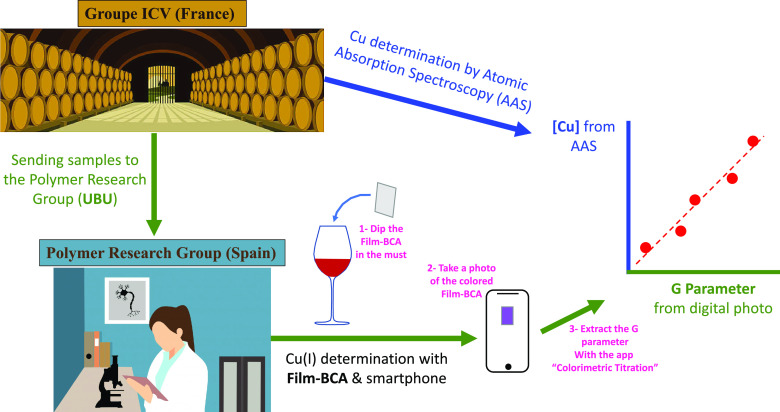
Graphical abstract of the experimental procedure carried out for the quantification of copper in grape must samples by FAAS and by using **Film-BCA**.

Similarly, to the procedure described in Section 2.8, 18 grape must samples from the French company Groupe ICV were measured in duplicate by mixing 2 mL of grape must with 50 μL of an aqueous solution of ascorbic acid (0.08 g/mL) buffered at pH 5. The sensory squares (8 mm side) were finally dipped in this solution for 12 h, washed with water (10 mL for 15 min) and methanol (10 mL for 15 min), and washed again with water (10 mL for 15 min). Afterward, we photographed the sensory squares and extracted the G parameter from the digital images. This parameter was correlated with the results obtained from Groupe ICV by FAAS. Figure 2 shows the procedure schematically.

### 2.10 Limits of Detection (LOD) and Quantification (LOQ)

The limit of detection (LOD) and the limit of quantification (LOQ) of our sensory system were calculated by the following equations: LOD = 3.3 × SD/*s* and LOQ = 10 × SD/*s*, respectively, where “SD” is the standard deviation of the blank sample and “*s*” is the slope of a calibration curve in the region of a low analyte content, from 0.25 to 1 ppm. The calibration curve and the fitted equation are shown in Figure S6, Section S5.

## 3 Results and Discussion

In this work, we report on a new way to measure copper in the pre-fermentation stage of wine production. The process is carried out with a smart film-shaped sensory polymeric material able to interact with Cu(I), signaling this interaction by providing a color change. Below, we will briefly describe the design of the material, the interaction with Cu(I), and the proof of concept carried out with 18 grape musts.

### 3.1 Design of the Film-Shaped Smart Sensory Polymer

The study stems from the need expressed by the French company Groupe ICV to create an alternative method to FAAS as the most common method for measuring total copper in grape musts. Installing a flame photometer within each winery is an economically unfeasible strategy for highly delocalized companies such as Groupe ICV, which analyzes samples from branches distributed throughout the French territory. In addition, not all wineries have the appropriate facilities and specialized personnel to carry out copper quantification using the methods described in the literature,^27^ which require advanced equipment and handling of reagents/solvents. Therefore, we proposed simplifying the analysis as much as possible for this study using a sensory polymer and a smartphone.

The material is oriented to *in situ* field tests, and probably to be used by non-specialized personnel, so we designed a material resistant to careless handling containing a high mol % of MMA (49.75 mol %), a monomer that provides great rigidity to the material. On the other hand, the material needs to absorb the grape must sample (aqueous medium), so it also requires certain hydrophilicity, which in this case is provided by the VP monomer (49.75 mol %). Finally, the chemical modification of a molecule widely known in the field of copper detection, BCA, was proposed.^27^ This molecule cannot be chemically anchored to a polyacrylic material since it is not a monomer, so a polymerizable group was introduced into the chemical structure, as graphically depicted in Figure 1a. A small amount (0.5 mol %) of the resulting sensory monomer was copolymerized with VP and MMA but enough for a visual color change of the material in the presence of Cu(I).

The designed films are thermostable materials, chemically cross-linked with ethyleneglycol dimethacrylate (Figure 1b). The nominal cross-linking ratio of the material is 167, which is directly related to the water swelling percentage of the material (75%), as seen analogously in previous works.^31^**Film-BCA** had a glass transition temperature of 142 °C, a thermal resistance, in terms of 5 and 10% weight losses, of 351 and 370 °C, respectively, and Young’s modulus of 299 MPa.

### 3.2 Study of the Interaction of the Sensory Monomer **Mono-BCA** with Cu(I)

The stoichiometry of the **Mono-BCA**:Cu(I) complex was studied by mass spectrometry and UV–vis spectrophotometry, and the measurements were carried out by using DMSO as the solvent due to the low solubility of **Mono-BCA** both in water and water:organic solvent mixtures.

In the results from mass spectrometry, we found two different peaks related to two complexes with stoichiometries of 1:1 and 2:1 (**Mono-BCA**:Cu(I)), being the 2:1 stoichiometry the most expected one as described in similar studies carried out with BCA and Cu(I) (see Figure S4 in Section S4).^26^

**Figure 3:**
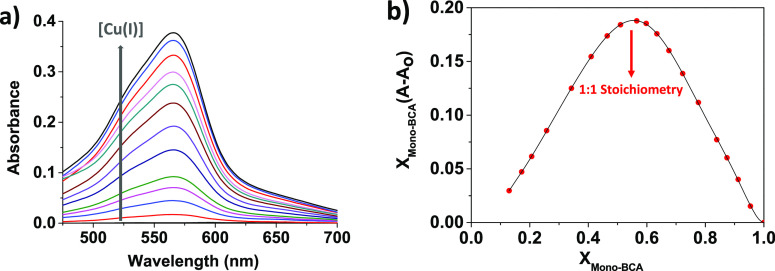
(a) Titration of Cu(I) with **Mono-BCA** in DMSO. The concentration of Cu(I) in the cuvette was increased from 4.9 × 10^–6^ to 4.1 × 10^–4^ M, and the initial concentration of **Mono-BCA** was 8.6 × 10^–5^ M. (b) Job’s plot diagram, which represents *X*_**Mono-BCA**_(*A* – *A*_0_) versus the molar fraction of **Mono-BCA**. “*A*_0_” is the initial absorbance at 566 nm ([Cu(I)] = 0 M), and “*A*” is the absorbance at 566 nm for each point of the titration.

Concerning UV–vis spectrophotometry, we focused on the band centered at 566 nm, corresponding to the formation of the colored complex, as shown in Figure 3a. Fitting of the titration profile provides a high complex formation constant, 3.6 × 10^6^ M, and the Job’s plot representation shows a maximum at **Mono-BCA**’s molar ratio of 0.5, which means a 1:1 stoichiometry (Figure 3b).

The experimental conditions, especially the solvent (DMSO), do not correspond to the real measurement conditions in grape musts (aqueous medium). Nevertheless, the results indicate a great affinity between **Mono-BCA** and Cu(I), so we extrapolate this behavior when working with **Film-BCA**.

### 3.3 Selectivity Study of **Film-BCA** with 30 Cations

**Figure 4:**
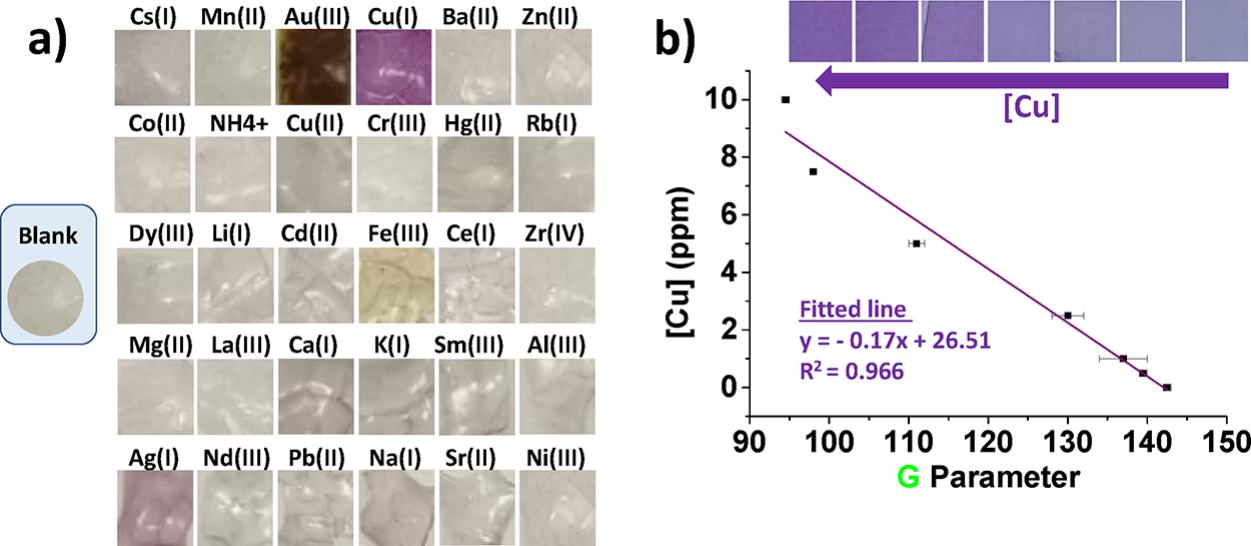
(a) Photograph of the qualitative experiment for testing the colorimetric response of **Film-BCA** with 30 cations. Experimental conditions: cation concentration = 5 × 10^–3^ M in pH 5 aqueous buffer; volume = 2 mL, dipping time = overnight. (b) Sensory squares (8 mm side) of **Film-BCA** were dipped for 12 h in aqueous solutions containing 2 mL of pH 5 buffered aqueous Cu(II) solutions (concentrations ranging from 0.5 to 10 ppm) and 50 μL of pH 5 buffered aqueous solution of ascorbic acid (0.08 g/mL). The graph shows the representation of the copper concentration vs the G parameter from the photographed squares (mean ± standard error of two replicates).

As shown in Figure 4a, **Film-BCA** only changes color in the presence of Cu(I) and Ag(I). Therefore, **Film-BCA** does not change color with the most abundant cations in wine, such as Na(I) or K(I), which is a great advantage. In addition, Ag(I) concentrations in wine are negligible,^32,33^ so we consider that **Film-BCA** does not present interferences to quantify copper in grape musts.

### 3.4 Preliminary Experiments: Copper Quantification in Aqueous Solutions Using **Film-BCA**

After dipping squares of **Film-BCA** in Cu(I) solutions with concentrations ranging from 0.5 to 10 ppm, and after taking a photograph of the films, the G parameter was extracted and represented against the copper concentration. The trend is linear in this concentration range, as shown in Figure 4b, and the obtained limits of detection (LOD) and quantification (LOQ) were 0.08 and 0.25 ppm, respectively (more information in Section S5). Therefore, these limits are suitable for this application, where the most found copper concentrations range is 0.2–6 ppm.

### 3.5 Proof of Concept: Copper Quantification in Grape Musts Using **Film-BCA**

**Figure 5:**
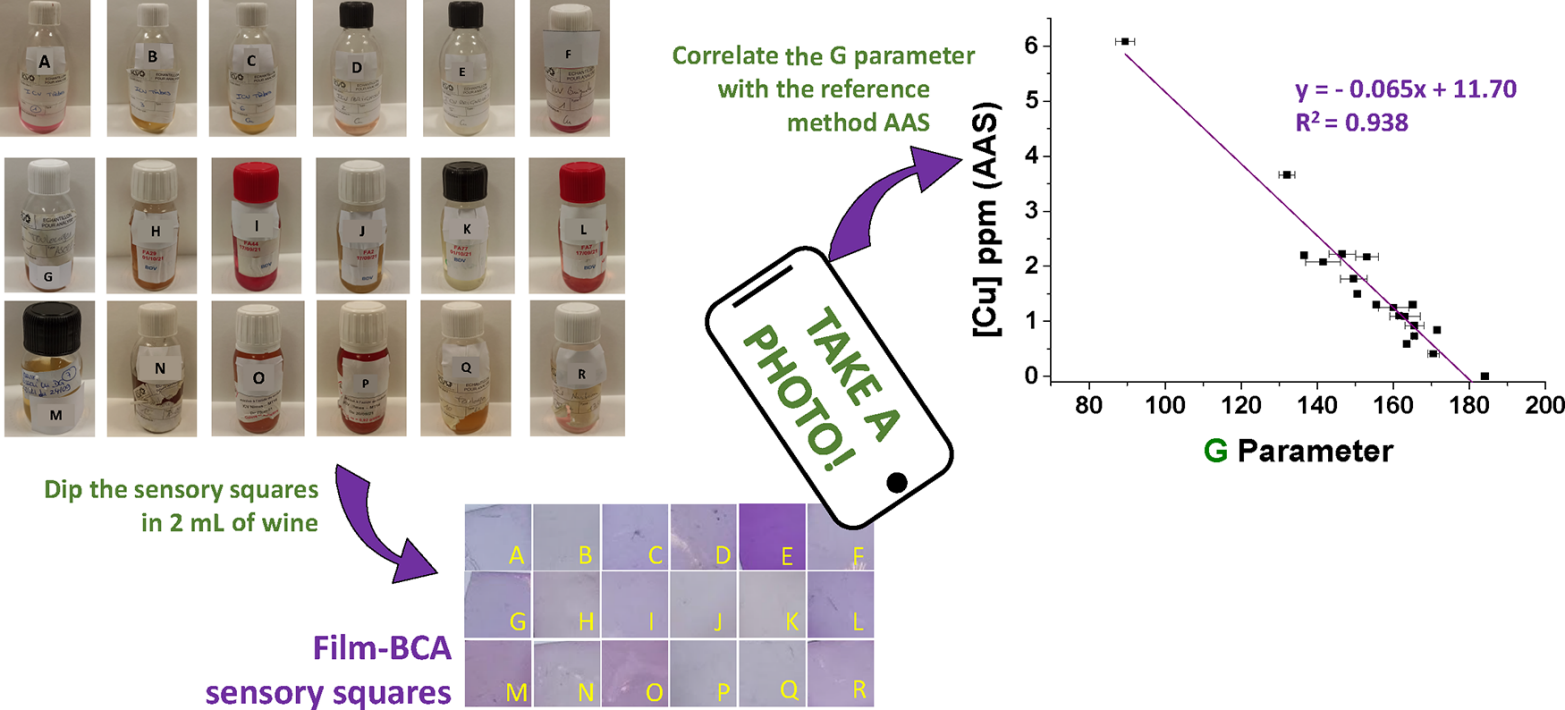
Graphical abstract of the proof of concept, including the representation of the Cu concentration obtained by the reference method (FAAS) against the G parameter obtained from the photographs (**Film-BCA**, data are means ± standard error of two replicates).

After validating the operation of **Film-BCA** in preliminary tests, we set out to carry out a proof of concept using real samples of grape must from the wine industry. The copper concentration results obtained by Groupe ICV in France were used as a reference (FAAS), so the color obtained in the **Film-BCA** sensor squares was plotted against those reference data. The result of this fitting is shown in Figure 5. Finally, if the G parameter of each sample is reintroduced in that fitted equation, then the copper concentration data shown in Table 1 are obtained and these results can be compared with the reference method.

**Table 1:** Cu(I) Concentration of Measured Grape Musts Obtained by the Reference Method (FAAS) and the Proposed Method, Based on the Use of **Film-BCA** and a Smartphone^a^

	Cu concentration (ppm)		
sample ID	geographical origin	FAAS	**Film-Cu**
A	Trèbes	0.73	0.89 ± 0.03
B	Trèbes	0.59	1.02 ± 0.03
C	Trèbes	1.3	1.54 ± 0.03
D	Provence	2.08	2.45 ± 0.29
E	Provence	6.08	5.85 ± 0.16
F	Provence	1.09	1.05 ± 0.26
G	Toulouges	1.5	1.87 ± 0.03
H	Beaumes	1.25	1.25 ± 0.26
I	Beaumes	2.17	1.70 ± 0.20
J	Beaumes	0.84	0.49 ± 0.03
K	Beaumes	0.41	0.56 ± 0.10
L	Beaumes	2.22	2.13 ± 0.23
M	Maurin	3.66	3.08 ± 0.13
N	Toulouges	1.1	1.15 ± 0.03
O	Nîmes	2.2	2.78 ± 0.03
P	Nîmes	0.92	0.89 ± 0.16
Q	Toulouges	1.3	0.93 ± 0.07
R	Narbonne	1.77	1.93 ± 0.23

a The Cu concentration data from the **Film-Cu** method are means ± standard errors of two replicates.

The data shown in Table 1 have been statistically analyzed with a Mann–Whitney independent sample *t*-test (non-parametric test; Section S6), and we can affirm that there is no significant difference between the means of the two methods.

Samples B and J require special mention since the FAAS method indicates that sample J has more copper, and method B indicates that sample B is the most concentrated. Our interpretation is that there was some contamination when carrying out the FAAS sample preparation, something relatively acceptable in an agricultural environment.

In short, our proposed material can be easily used at wineries to analyze copper in grape musts. This could represent a significant advance for wineries since the timeouts and sample transport are reduced, increasing productivity and reducing the risk of cupric cracks, with the only technical requirement of having a smartphone. Comparatively, Table 2 shows the most used methods for the quantification of copper, especially in wineries, emphasizing the possibility of carrying out an *in situ* detection, with the required equipment, and the detection limits.

**Table 2:** Figure of Merits Showing the Advantages and Disadvantages of the Proposed Methodology for the Quantification of Cu in Grape Musts/Wines against the Most Used Ones

detection method	*in situ* measure	quantification in wine/grape must	equipment required	LOD	ref
FAAS	no	yes	flame atomic absorption spectrometer	0.40 μg/L	(34)
ICP-MS	no	no	ICP-mass spectrometer	2.0 μg/g	(35)
	no	yes	ICP-mass spectrometer		(36)
	no	yes	ICP-mass spectrometer	0.04 μg/L	(37)
	no	yes	ICP-mass spectrometer	0.6 μg/L	(38)
fluorimetry probes in solution	no	no	fluorimeter		(39)
	no	yes	fluorimeter	8.2 μg/L	(40)
total reflection X-ray fluorescence	no	yes	TXRF spectrometer	0.1 mg/L	(41)
colorimetric probes in solution	no	yes	UV–vis spectrophotometer	0.01 mg/L	(42)
	no	no	UV–vis spectrophotometer	0.5 nM	(43)
colorimetric chemosensor nanofibrous hydrogel	yes	no	UV–vis spectrophotometer	0.01 mg/L	(44)
colorimetric film (**Film-BCA**)	yes	yes	smartphone	0.08 mg/L	this work

## 4 Conclusions

*In situ* and inexpensive detection methods and methodologies are tools that are in great demand by companies dedicated to laboratory analysis, especially when results are needed quickly and sample sending is ruled out. In collaboration with the company Groupe ICV, dedicated to chemical analysis in the wine industry, we have jointly developed a film-shaped smart sensory polymer for the quantification of copper in grape must, the novelty of which is that it does not require specialized personnel, reagents, or specialized equipment. The material is an excellent tool for this type of company, which has wineries all over the country and cannot afford the installation and maintenance of expensive and advanced equipment for quantifying a key parameter in wine production, such as the copper concentration. It is a robust system that we have tested with real samples of grape musts provided by the mentioned company, which are also from different growing areas, such as Narbonne, Nîmes, Maurin, Beaumes, Toulouges, Provence, and Trèbes. For this publication, we have worked with dipping times of 12 h since it is important to reach the system’s equilibrium to draw solid conclusions. However, this time can be reduced and adapted to the needs of the industry. The limits of detection (LOD) and quantification (LOQ) offered by the material are 0.08 and 0.25 ppm, respectively, which makes it suitable for this specific application. The sensory material, methodology, and necessary equipment have been easily oriented for use by non-specialized personnel, and therefore, the only device needed for copper quantification is a smartphone.

## Supporting Information Available

Information about grape must samples; synthesis and characterization of **Mono-BCA**; characterization of the sensory polymer **Film-BCA**; high-resolution mass spectra of the 2:1 and 1:1 **Mono-BCA**:Cu(I) complexes; information about the limit of detection (LOD) and limit of quantification (LOQ); statistical analysis (PDF)

## Notes

Open Data is available at https://riubu.ubu.es/handle/10259/5684 under the name “Democratization of copper analysis in grape must following a polymer-based lab-on-a-chip approach”.
