# Supplementary material for: Democratization of Copper Analysis in Grape Must Following a Polymer-Based Lab-on-a-Chip Approach
Source: ACS Appl Mater Interfaces. 2023 Mar 20;15(12):16055–62. doi: 10.1021/acsami.3c00395 (PMC10064320; doi:10.1021/acsami.3c00395)
Supplement: Supplementary file 1 — am3c00395_si_001.pdf [file am3c00395_si_001.pdf]

Democratization of copper analysis in grape must following a polymer-based lab-on-a-chip approach

José Carlos Guirado-Moreno[a], Israel Carreira-Barral[a], Saturnino Ibeas[b], José M. García[a], Daniel Granès[b], Nicolas Marchet[b], Saúl Vallejos[a,*]

[a] Departamento de Química, Facultad de Ciencias, Universidad de Burgos, Plaza Misael Bañuelos s/n, 09001 Burgos, Spain
*Corresponding author: Dr Vallejos (svallejos@ubu.es)

Table of contents

S1. Grape must samples 2
S2. Synthesis and characterization of Mono-BCA 8
S3. Characterization of the sensory polymer Film-BCA 10
S4. High-resolution mass spectra of the 2:1 and 1:1 Mono-BCA:Cu(I) complexes 11
S5. Limit of detection (LOD) and limit of quantification (LOQ) 13
S6. Statistical analysis. Comparison between Film-Cu and flame atomic absorption spectrometry (FAAS) methods 14

S1. Grape must samples

Table S1. Images of the 18 must samples sent by *ICV Groupe* to the *Polymer Research Group* at the University of Burgos. The table also indicates the sample ID, the geographical origin, the photograph of **Film-BCA** sensory squares after the analysis, and the green (G) parameter from RGB digital color space obtained from photographs (data means of ± standard errors of 2 replicates). Experimental conditions for the analysis: the 8 mm side squares were dipped for 12 hours in a solution of 2 mL of must and 50 µL of an aqueous solution of ascorbic acid (0.08 g/mL) buffered at pH 5.

Sample ID	Geographical origin	Images	Film-BCA	
			Photograph	Extracted G parameter
A	Trèbes	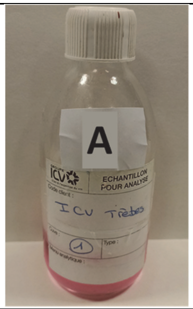	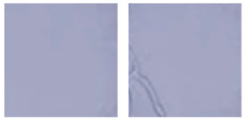	165.5 ± 0.5
B	Trèbes	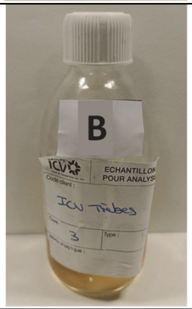	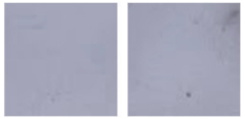	163.5 ± 0.5
C	Trèbes	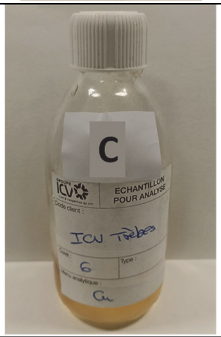	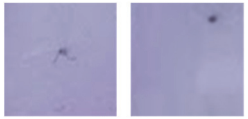	155.5 ± 0.5

D	Provence	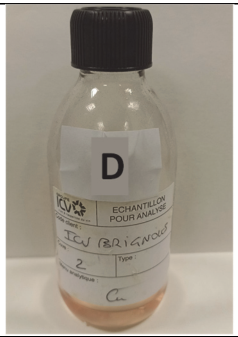	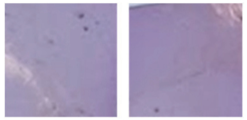	141.5 ± 4.5
E	Provence	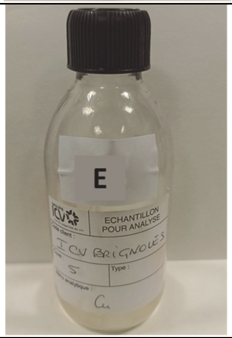	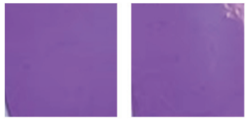	89.5 ± 2.5
F	Provence	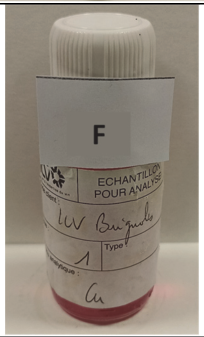	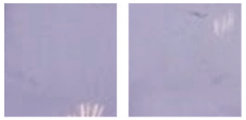	163 ± 4

G	Toulouges	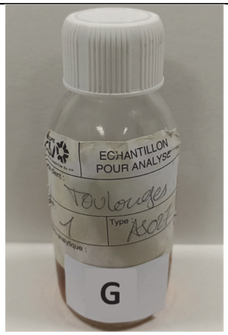	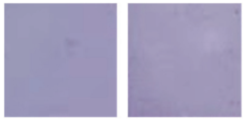	150.5 ± 0.5
H	Beaumes	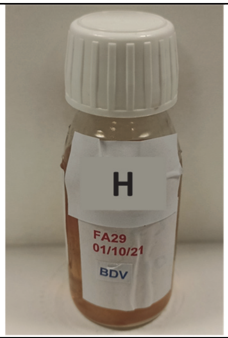	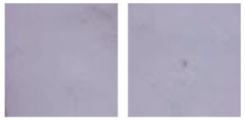	160 ± 4
I	Beaumes	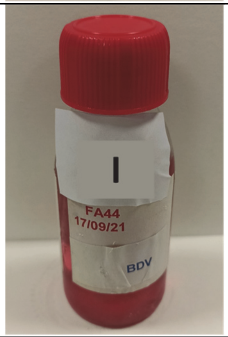	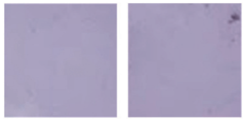	153 ± 3

J	Beaumes	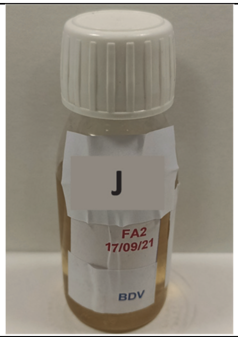	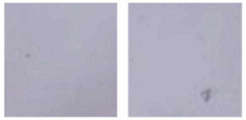	171.5 ± 0.5
K	Beaumes	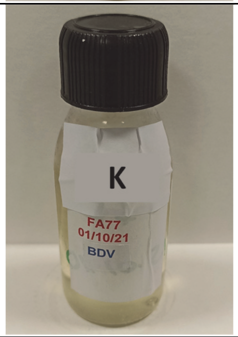	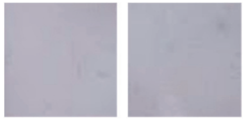	170.5 ± 1.5
L	Beaumes	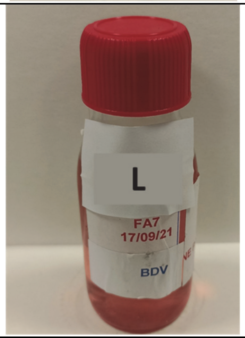	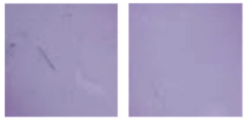	146.5 ± 3.5

M	Maurin	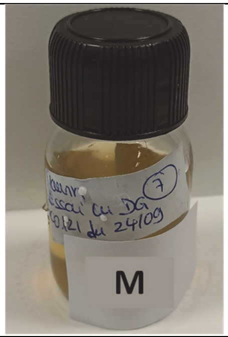	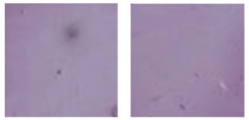	132 ± 2
N	Toulouges	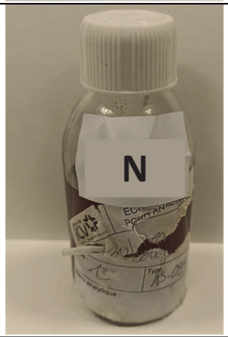	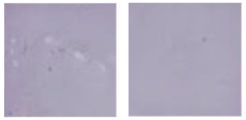	161.5 ± 0.5
O	Nîmes	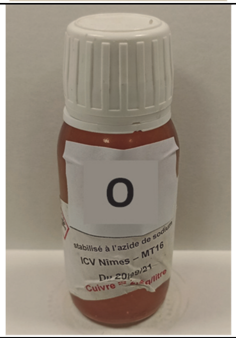	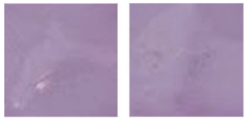	136.5 ± 0.5

P	Nîmes	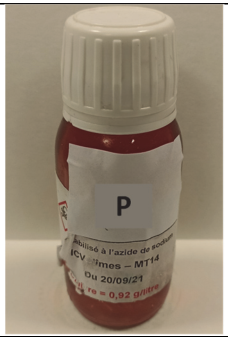	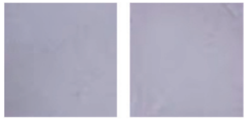	165.5 ± 2.5
Q	Toulouges	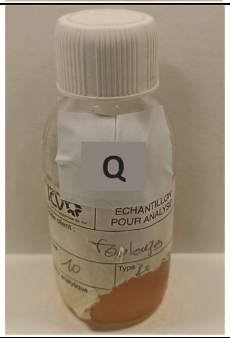	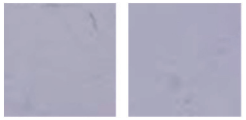	165 ± 1
R	Narbonne	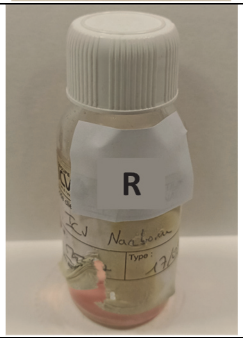	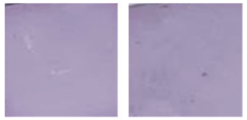	149.5 ± 3.5

S2. Synthesis and characterization of Mono-BCA

- ***Synthesis of BCA***

BCA synthesis was performed as described by Aizenberg *et al.* [1]. Firstly, Bicinchoninic acid disodium salt (2.05 g, 4.88 mmol) was dissolved in 35 mL DMF. Secondly, 5 mL of HCl 2 M was added with stirring. The resultant mixture was stirred at room temperature overnight. After that, the solid was filtered, and washed with water and acetone. (Yield: 52%, 0.87g). ^{1}H NMR (300 MHz, DMSO-d_6) δ (ppm): 14.04 (s, 2H), 9.11 (s, 2H), 8.75 (d, J = 8.5 Hz, 2H), 8.24 (d, J = 8.4 Hz, 2H), 7.86 (t, J = 7.3 Hz, 2H), 7.72 (t, J = 7.7 Hz, 2H). ^{13}C NMR (75 MHz, DMSO-d_6) δ (ppm): 167.91, 154.68, 148.58, 137.57, 130.72, 130.56, 129.20, 126.10, 125.30, 119.88. HRMS m/z [M+H]+ calc.: 345.0831; found: 345.0865.

a)

b)

c)

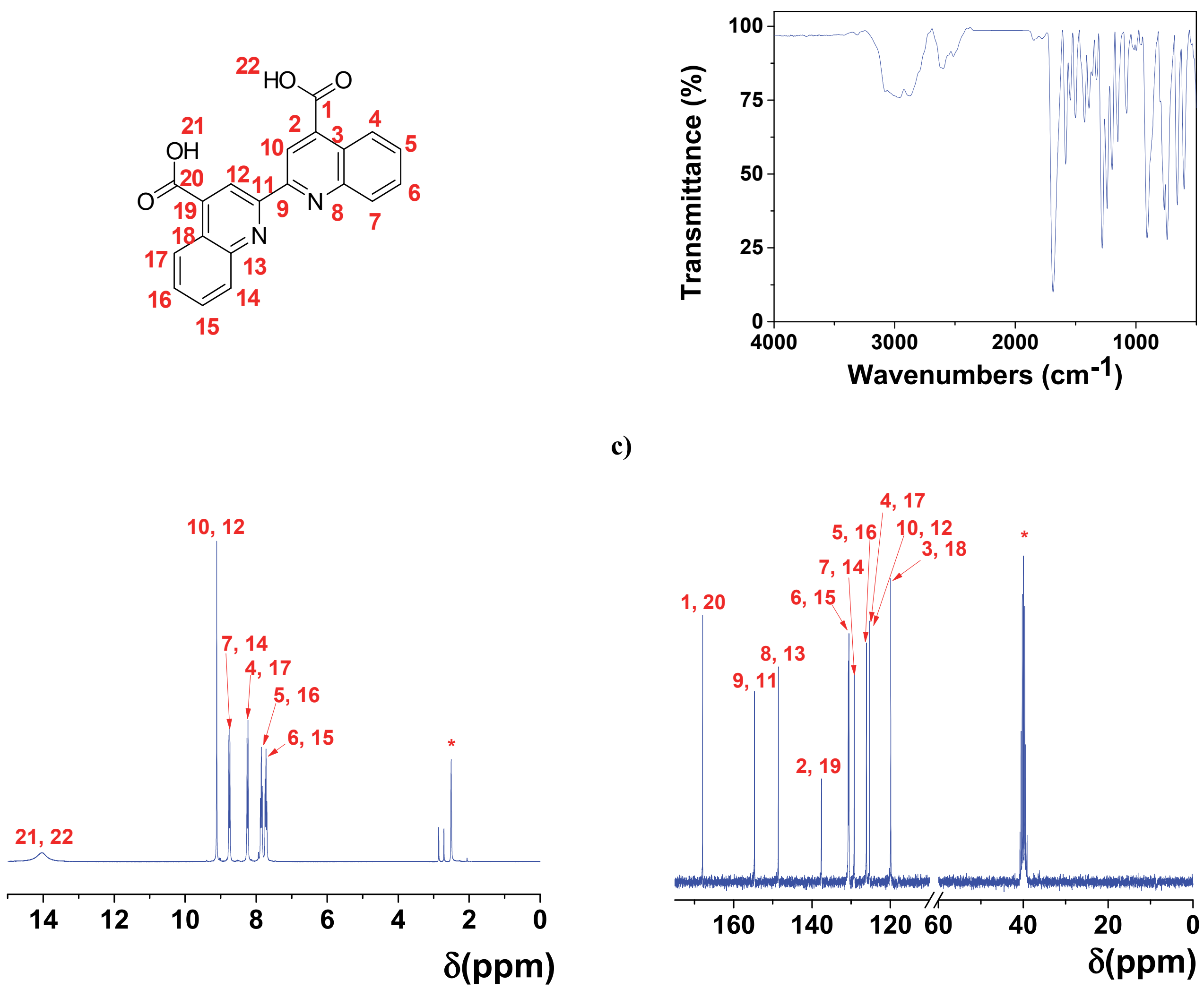

Figure S1. Characterization of BCA by: (a) FTIR; (b) ^{1}H NMR; and (c) ^{13}C NMR (* = solvent signal, DMSO-d_6).

- ***Synthesis of N4,N4'-bis(4-vinylphenyl)-[2,2'-biquinoline]-4,4'-dicarboxamide (Mono-BCA)***

Mono-BCA synthesis was performed as described by Aizenberg *et al.* [1]. **Mono-BCA** was prepared by refluxing BCA overnight (0.51 g, 1.48 mmol) in 8 mL of thionyl chloride. The product was dried under vacuum and was dissolved in 10 mL of THF immediately for next step reaction. Then, a solution of 4-animostyrene (0.62 g, 5.21 mmol) and triethylamine (0.64 g, 6.38 mmol) in 10 mL of THF was added dropwise under N_2. The mixture was diluted with 5 mL of THF and stirred overnight at room temperature. Finally, the suspension was filtered and washed with THF (25 mL), 3% ammonia solution (10mL) and water (15mL). (Yield: 0.755g, 86%) ^{1}H NMR (300 MHz, DMSO-d_6) δ (ppm): 11.09 (s, 2H), 8.98 (s, 2H), 8.33 (s, 2H), 8.22 (s, 2H), 7.94 (s, 2H), 7.84 (s, 4H), 7.78 (s, 2H), 7.57 (s, 4H), 6.71 (s, 2H), 5.85 (s, 2H), 5.27 (s, 2H). ^{13}C NMR (75 MHz, DMSO-d_6) δ (ppm): 165.72, 154.81, 148.15, 143.91, 138.90, 136.61, 133.70, 131.28, 130.44, 129.13, 127.20, 125.84, 124.91, 120.59, 116.77, 113.89. HRMS *m/z* [M+H]+ calc.: 547.2089; found: 547.2122.

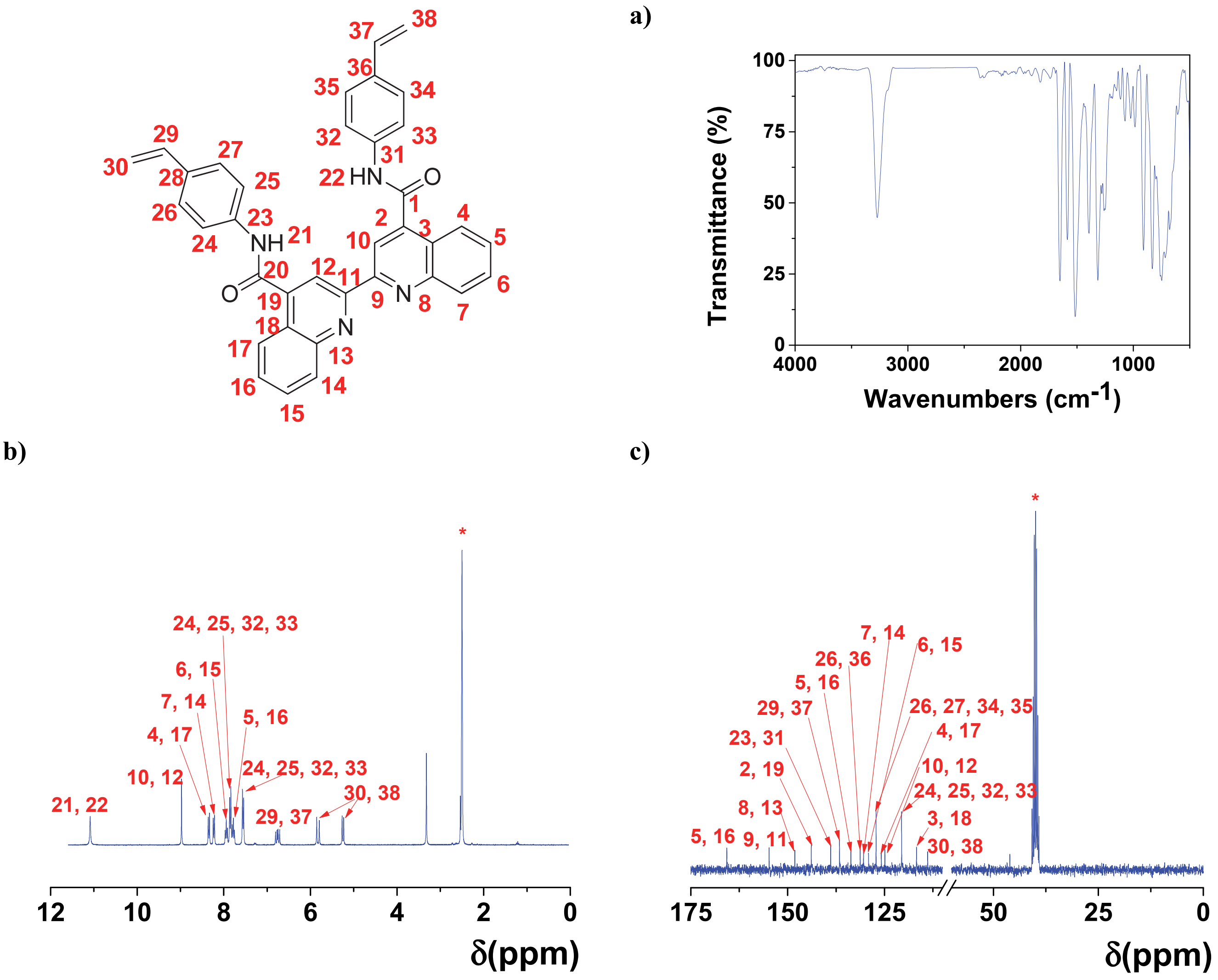

Figure S2. Characterization of **Mono-BCA** by: (a) FTIR; (b) ^{1}H NMR; and (c) ^{13}C NMR (* = solvent signal, DMSO-d_6).

S3. Characterization of the sensory polymer Film-BCA

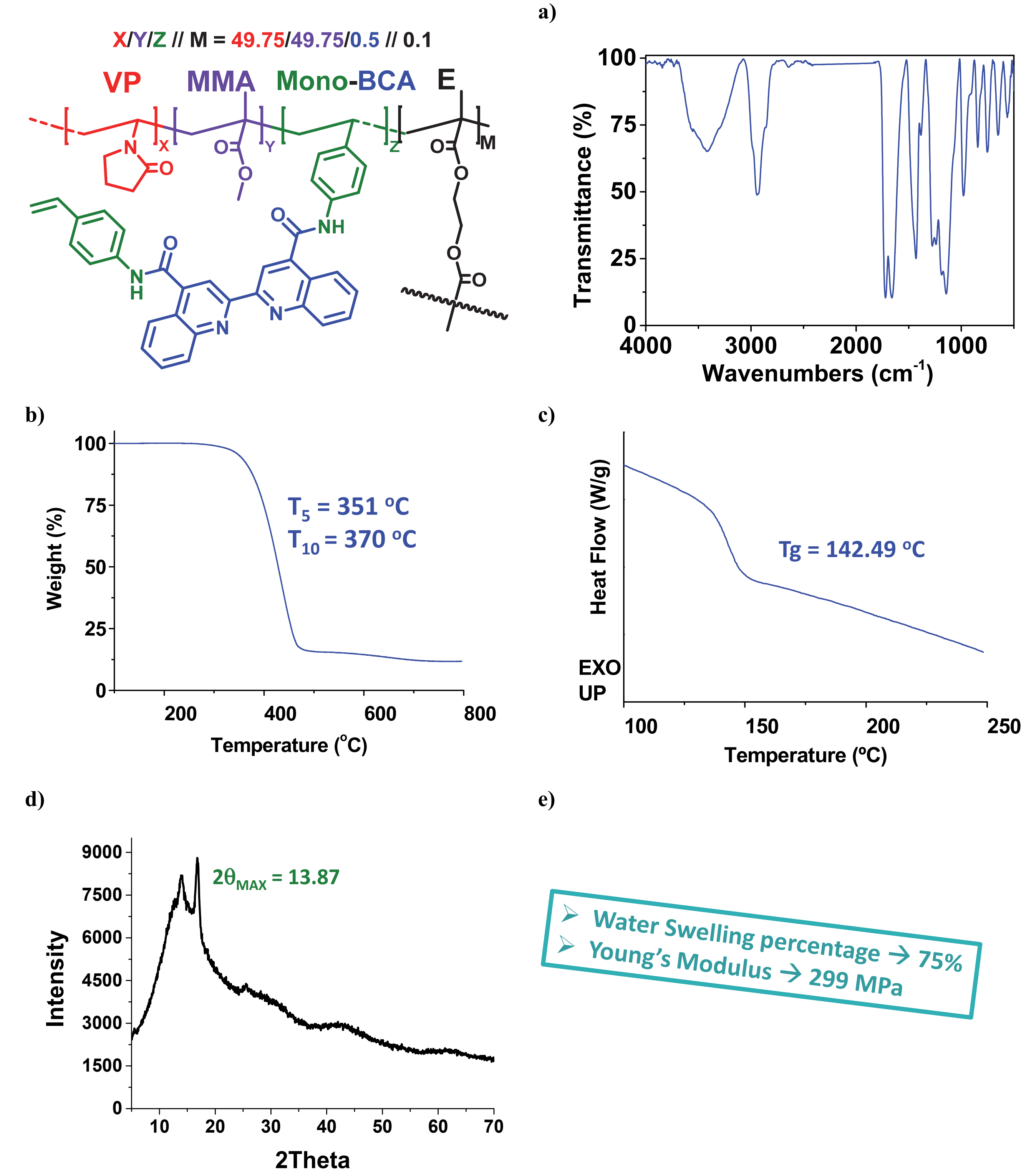

Figure S3. Characterization of the film-shaped copolymers **Film-BCA**: **(a)** FT-IR spectra of the polymer; **(b)** thermogravimetric curves at 10°C·min^{-1} under nitrogen atmosphere showing T_5 and T_{10} temperatures; **(c)** DSC curve at a heating rate of 20 °C·min^{-1} under nitrogen atmosphere showing Tg value; **(d)** PDRX of the **Film-BCA**; **(e)** additional characterization data.

S4. High-resolution mass spectra of the 2:1 and 1:1 Mono-BCA:Cu(I) complexes

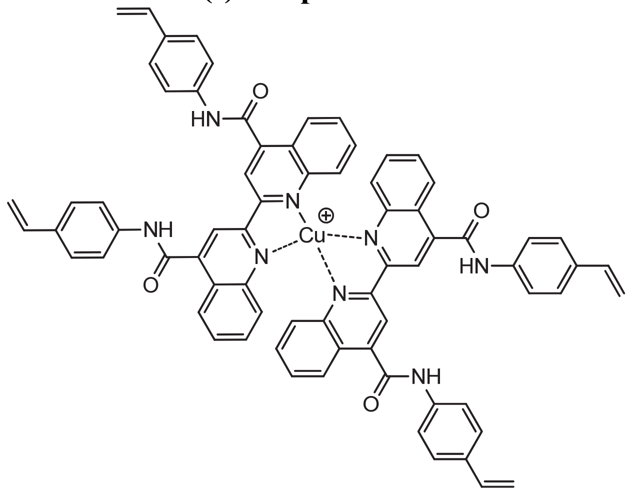

a)

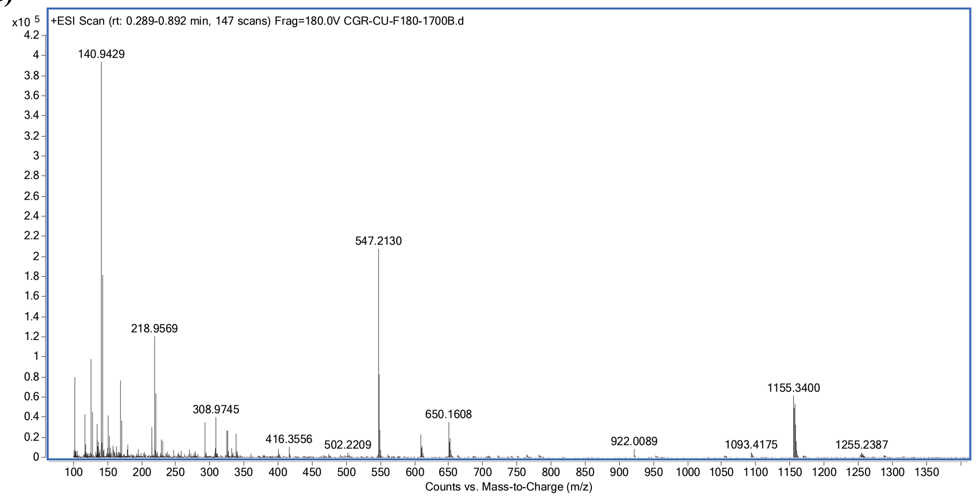

b)

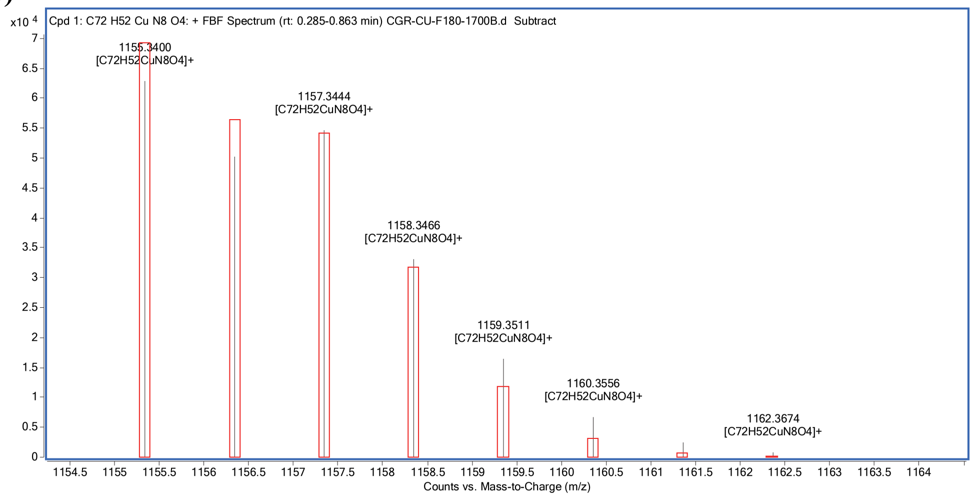

HRMS (+ESI): found *m/z* = 1155.3405 $[C_{72}H_{52}CuN_8O_4]^+$; calculated *m/z* = 1155.3402

Figure S4. Characterization of the **Mono-BCA**:Cu(I) complex with 2:1 stoichiometry (proposed chemical structure of the molecular ion is shown above): (a) high resolution mass spectrum of the sample; (b) magnification of the peak corresponding to the 2:1 complex, showing the found mass (black lines) and the calculated mass (reddish rectangles).

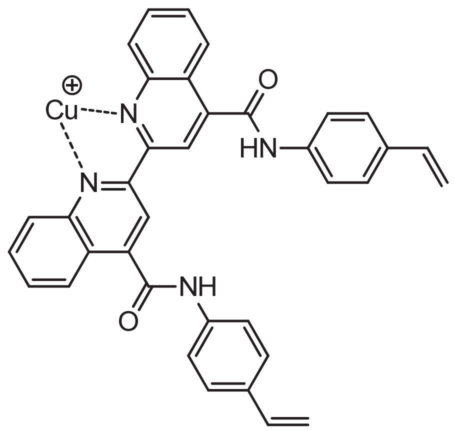

a)

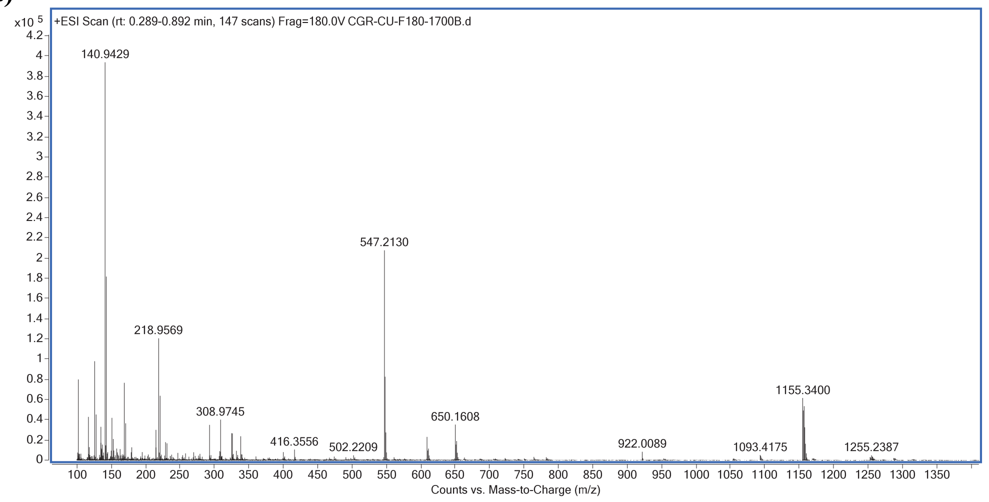

b)

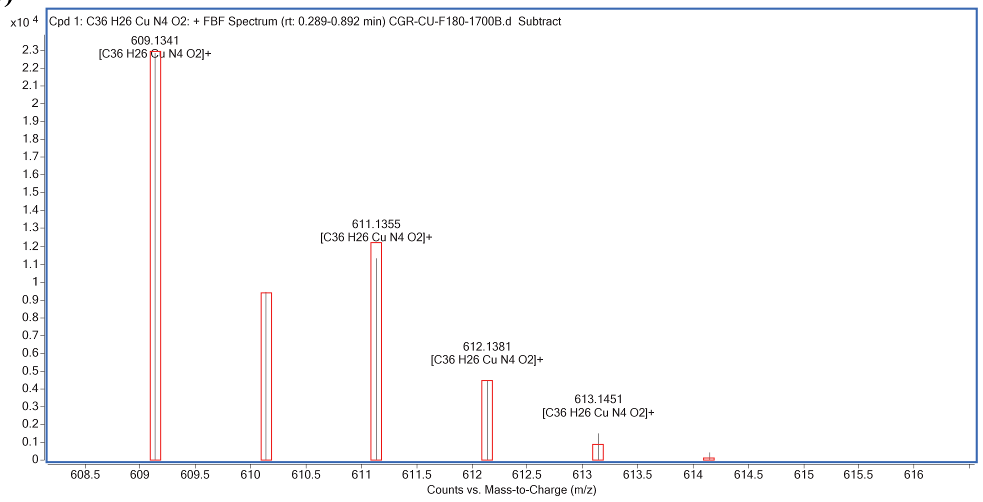

HR-MS (+ESI): found m/z = 609.1341 $[C_{36}H_{26}CuN_4O_2]^+$; calculated m/z = 609.1346.

Figure S5. Characterization of the **Mono-BCA**:Cu(I) complex with 1:1 stoichiometry (proposed chemical structure of the molecular ion is shown above): (a) high resolution mass spectrum of the sample; (b) magnification of the peak corresponding to the 1:1 complex, showing the found mass (black lines) and the calculated mass (reddish rectangles).

S5. Limit of detection (LOD) and limit of quantification (LOQ)

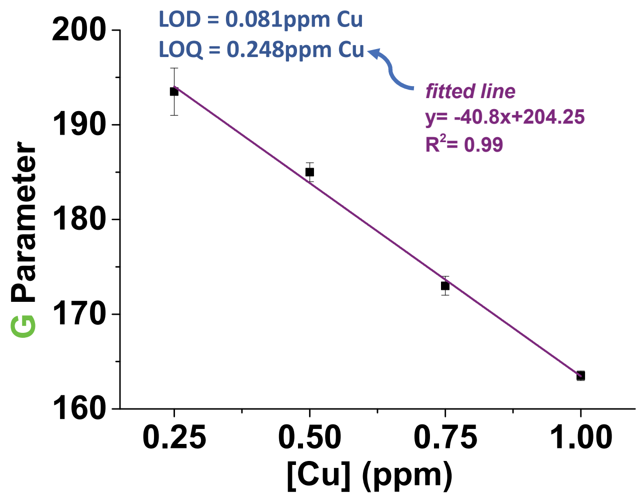

Figure S6. Graphical representation of the G parameter of the photographed squares against Cu concentration. Data are means ± standard error of 2 replicates. The titration is performed at low concentrations to calculate the LOD and LOQ through the following equations: LOD=3.3 × *SD* / *s* and LOQ=10 × *SD* / *s*, respectively, where *SD* is the standard deviation of the blank sample and *s* is the slope of the fitted line.

S6. Statistical analysis. Comparison between Film-Cu and flame atomic absorption spectrometry (FAAS) methods.

This procedure is designed to compare two data samples and will determine if there are statistically significant differences between the two methods.

A) Description of the variables

First, we checked the normal distribution of the data. **Table S2** contains the statistical summary for the two data samples. Since the P-values of Shapiro-Wilk are less than 0.05, both methods do not come from normal distributions, so we must carry out a Mann-Whitney independent samples t-test (non-parametric test).

Table S2. Statistical Summary

	**FAAS**	**Film-Cu**
**Count**	18	18
**Mean**	1.753	1.734
**Standard deviation**	1.264	1.337
**Shapiro-Wilk**	0.792	0.762
**P-Value of Shapiro-Wilk**	0.001	< .001
**Maximum**	5.850	6.080
**Minimum**	0.490	0.410

B) Means comparison

The Mann-Whitney independent samples t-test evaluates specific hypotheses about the difference between the means of the two methods, which do not have normal distributions. In our case, hypotheses are:

→ Ho: There is no difference between the means
→ Ha: There is a difference between the means
→ P-value = 0.975

Since the calculated P-value is greater than 0.05, the null hypothesis cannot be rejected for alpha = 0.05, and therefore **there is no difference between the means of the two methods.**

References

[1] M. Aizenberg, K. Okeyoshi, J. Aizenberg, Inverting the Swelling Trends in Modular Self-Oscillating Gels Crosslinked by Redox-Active Metal Bipyridine Complexes, Adv. Funct. Mater. 28 (2018). https://doi.org/10.1002/adfm.201704205.
